# Real-time visualization of renal microperfusion using laser speckle contrast imaging

**DOI:** 10.1117/1.JBO.26.5.056004

**Published:** 2021-05-22

**Authors:** Wido Heeman, Hanno Maassen, Joost Calon, Harry van Goor, Henri Leuvenink, Gooitzen M. van Dam, E. Christiaan Boerma

**Affiliations:** a University of Groningen, Faculty Campus Fryslân, Leeuwarden, The Netherlands; b University Medical Centre Groningen, Department of Surgery, Groningen, The Netherlands; c LIMIS Development BV, Leeuwarden, The Netherlands; d University Medical Centre Groningen, Department of Pathology and Medical Biology, Groningen, The Netherlands; e ZiuZ Visual Intelligence, Gorredijk, The Netherlands; f Medical Centre Leeuwarden, Department of Intensive Care, Leeuwarden, The Netherlands

**Keywords:** laser speckle contrast imaging, transplantation, kidney, sidestream dark-field imaging, renal microperfusion

## Abstract

**Significance:** Intraoperative parameters of renal cortical microperfusion (RCM) have been associated with postoperative ischemia/reperfusion injury. Laser speckle contrast imaging (LSCI) could provide valuable information in this regard with the advantage over the current standard of care of being a non-contact and full-field imaging technique.

**Aim:** Our study aims to validate the use of LSCI for the visualization of RCM on *ex vivo* perfused human-sized porcine kidneys in various models of hemodynamic changes.

**Approach:** A comparison was made between three renal perfusion measures: LSCI, the total arterial renal blood flow (RBF), and sidestream dark-field (SDF) imaging in different settings of ischemia/reperfusion.

**Results:** LSCI showed a good correlation with RBF for the reperfusion experiment (0.94±0.02; p<0.0001) and short- and long-lasting local ischemia (0.90±0.03; p<0.0001 and 0.81±0.08; p<0.0001, respectively). The correlation decreased for low flow situations due to RBF redistribution. The correlation between LSCI and SDF (0.81±0.10; p<0.0001) showed superiority over RBF (0.54±0.22; p<0.0001).

**Conclusions:** LSCI is capable of imaging RCM with high spatial and temporal resolutions. It can instantaneously detect local perfusion deficits, which is not possible with the current standard of care. Further development of LSCI in transplant surgery could help with clinical decision making.

## 1 Introduction

Intraoperative-hampered renal cortical microperfusion (RCM) during, for example, anastomosis has been associated with ischemia/reperfusion-injury related postoperative complications. Others have shown the potential of near-surface perfusion imaging for the prediction of postoperative complications, including reduced creatinine clearance, delayed graft function, and even graft rejection.^1^^–^^4^ As such, it is conceivable that intraoperative monitoring of RCM using optical imaging methods may support surgical decision making, which may lead to improved perfusion during organ reperfusion and potentially contribute to a reduction in unfavorable postoperative outcomes.

The value of the common techniques, such as postoperative duplex sonography or an arterial renal blood flow (RBF) probe, to monitor total RBF is limited by the fact that such techniques do not account for local perfusion heterogeneities.^5^ It is based on the misconception that total RBF adequately reflects RCM.^6^ Therefore, the use of techniques that can detect heterogeneity of blood flow is preferred.^7^ Postoperative duplex sonography has the ability to detect local perfusion deficits^8^ and has been validated in several studies. However, its general application is limited by substantial operator dependency.^5^ Others reported on the use of contact imaging methods to directly visualize and quantify red blood cell (RBC) movement.^2^^,^^3^ These methods yielded promising results regarding certain cut-off values for delayed graft function, postoperative creatinine levels, or even allograft rejection with RCM measurements performed as soon as 5 min after reperfusion.^1^^–^^4^ The main limitation of these methods is the small ($∼1\;{\mathrm{mm}}^{2}$) field-of-view (FOV) in which the RCM can be visualized. Recently, indocyanine green (ICG) fluorescence imaging has been introduced to assess the RCM and to correlate it to clinical outcome of kidney transplantation.^5^^,^^9^^,^^10^ However, ICG fluorescence is hard to quantify^10^ and the presence of a fluorescent signal does not immediately imply a well-perfused organ.^11^ The administration of the fluorescent dye, which is required each time the perfusion is measured, also hinders the surgical procedure.

To date, an objective, intraoperative imaging tool that can help visualize the RCM during surgery is still missing. In this paper, we report on the use of laser speckle contrast imaging (LSCI), a real-time, non-contact, full-field imaging technique with a large FOV that can visualize blood flow in tissues without the administration of a fluorescent dye,^12^ to monitor RCM in human-sized porcine kidneys. We aim to validate the use of LSCI as a tool for organ reperfusion measurement during several models of hemodynamic changes.

## 2 Materials and Methods

### 2.1 Slaughterhouse Kidneys

Six slaughterhouse-retrieved porcine kidneys were obtained from a local abattoir. Pigs (female Dutch Landrace pigs, approximately 5 months old with an average weight of 130 kg) were slaughtered for consumption purposes and were handled according to standardized legal procedures. The pigs were stunned by electricity and died from exsanguination. Approximately 2 liters of blood were collected in a beaker with 25,000 IU of heparin (LEO Pharma A/S, Ballerup, Denmark) during exsanguination. Kidneys were removed from the cadaver en bloc, the renal artery was dissected free, and surrounding tissue was removed. The left kidney was used for all the experiments since this side displayed a better visible arterial bifurcation. After 30 min of warm ischemia (i.e., the time between the stop of the circulation of blood and the start of the cold flush), the kidneys were flushed with 500 ml cold 4°C saline. Next, the kidneys were installed in a kidney holder and placed on the hypothermic machine perfusion (HMP) (Kidney Assist Transporter, Organ assist^®^, Groningen, The Netherlands) at 4°C and perfused for three and a half hours at a mean pressure of 25 mmHg. HMP was oxygenated (100% $O_{2}$) at a rate of 100  ml/min.

### 2.2 Normothermic Machine Perfusion

**Fig. 1:**
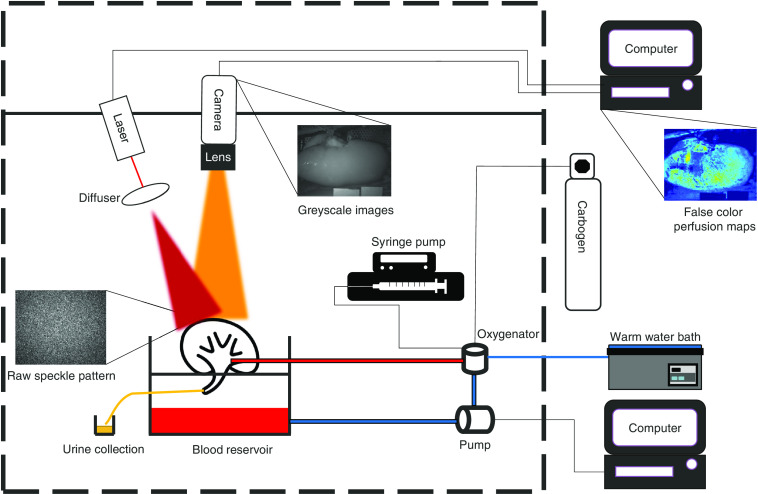
Schematic overview of the experimental setup which allows for simultaneous blood flow measurements using LSCI and total RBF. The LSCI setup consists of a grayscale camera with optics, a red 638-nm laser with a diffuser, and a computer to run the LSCI algorithm. The laser light homogenously illuminates the kidney, which allows for full-field, real-time perfusion imaging of the cortical blood flow. The kidney is connected to a computer that measures and regulates the total RBF, pressure, and temperature. The autologous blood is oxygenated using carbogen and warmed using a warm water bath. The syringe pump is used to add compounds to keep the blood composition stable. The urine is collected to monitor urine production.

The setup of the normothermic machine perfusion (NMP) was described in detail elsewhere,^13^ using a centrifugal pump head (Deltastream DP3^®^, MEDOS Medizintechnik AG, Heilbronn, Germany) controlled by in-house developed software (Sophistikate^®^, Labview, National Instruments, Austin, United States).^14^ The software not only enables both flow and pressure directed perfusion but also allows to switch between pulsatile sinusoidal and constant flow. The temperature was regulated using a Jubalo water heating system and set at 37°C. An integrated heat exchanger (HILITE 1000^®^, MEDOS Medizintechnik AG, Heilbronn, Germany) was built in the oxygenator. The flow sensor is a clamp-on flow sensor (ME7PXL clamp^®^, Transonic Systems Inc., Ithaca, United States). The pressure sensor is a Truewave^®^ disposable pressure transducer (Edwards Lifesciences, Irvine, Unites States). As perfusion medium, 500 ml of autologous leukocyte depleted blood was used. The blood was diluted with 300 ml Ringers lactate (Baxter, Utrecht, The Netherlands) and supplemented with 10 ml 8.4% bicarbonate (B. Braun Melsungen AG, Melsungen, Germany), 10 ml 5% glucose (Baxter, Utrecht, The Netherlands), 6 mg mannitol (Baxter, Utrecht, The Netherlands), 0.33 ml dexamethasone (Centrafarm, Etten-Leur, The Netherlands), 100  mg/200  mg amoxicilline/clavulanic acid (Sandoz B.V. Almere, The Netherlands), 90 mg creatinine (Sigma-Aldrich, St. Louis), and 0.1 ml sodium nitroprusside (Sigma-Aldrich, St. Louis). Plasma was added to reach a hematocrit of 24%. A constant infusion (20  ml/h) of a mixture of 90 ml Aminosol (Aminoplasmal, B. Braun Melsungen AG, Melsungen, Germany), 1 ml insulin (NovoRapid^®^, Novo Nordisk, Bagsværd, Denmark), and 3 ml bicarbonate was maintained. Carbogen (95% $O_{2}$ and 5% ${\mathrm{CO}}_{2}$) was supplied via the oxygenator at a flow rate of 500  ml/min. The complete NMP setup is shown in Fig. 1.

### 2.3 Laser Speckle Contrast Imaging Setup

LSCI is based on the principle of coherent laser light that is backscattered from tissue, which forms a speckle pattern at the detector. The phase shift of this backscattered light results in a random interference pattern, the so-called speckles. Due to motion in the tissue, i.e., movement of the RBCs, the interference pattern starts fluctuating, causing a dynamic speckle pattern, which is blurred by the finite exposure time of the detector. The speckle contrast K is calculated using Eq. (1).

$$K=\frac{\sigma }{\left\langle I\right\rangle },$$ (1)

where σ is the standard deviation of the intensity I over the mean intensity ⟨I⟩ calculated over a convolution window in space and/or time. An LSCI setup was built based on Lapvas^®^-imaging (LIMIS Development B.V., Leeuwarden, The Netherlands) analysis software that has previously been demonstrated by our group to qualify gut microvascular blood flow during *in-vivo* ischemia/reperfusion experiments.^15^ A monochrome camera (CM-200GE^®^, Jai, Copenhagen, Denmark) was placed on a fixed 3D-printed holder to ensure invariability in distance and incident angle of the camera and laser (Fig. 1). The distance between the camera and the kidney was 20 cm with a resulting FOV of 19×14  cm. The lens (LM12JC^®^, Kowa, Düsseldorf, Germany) was set at an f/number of 7 resulting in ∼2  pixels per speckle thus satisfying the Nyquist criterion.^16^ A polarizer filter was added to minimize specular reflections. Images were 1624×1236  pixels and were recorded with 3.125  frames/s and an exposure time of 40 ms. The longer exposure time is required in order to get adequate pixel intensities as a result of the combination of a low powered laser and large FOV. The images were analyzed using a time-averaged spatial LSCI algorithm with a spatial 7×7 sliding window and a temporal window of 7 frames. A red fiber-coupled laser diode (λ=638  nm, 200 mW; Lionix International, Enschede, The Netherlands) was coupled into an optical fiber with a collimating lens (12  mm Ø, −12  mm FL-uncoated double-concave lens, Edmund Optics, New Jersey, United States) at the distal end. The laser was mounted on a fixed bar and set to an output power of 120 mW. The total setup (Fig. 1) was placed in a blacked-out box to block all ambient light. 2D-perfusion maps were generated and displayed in real-time during the experiments while raw speckle images were stored for further offline postprocessing.

### 2.4 Hemodynamic Experiments

**Fig. 2:**
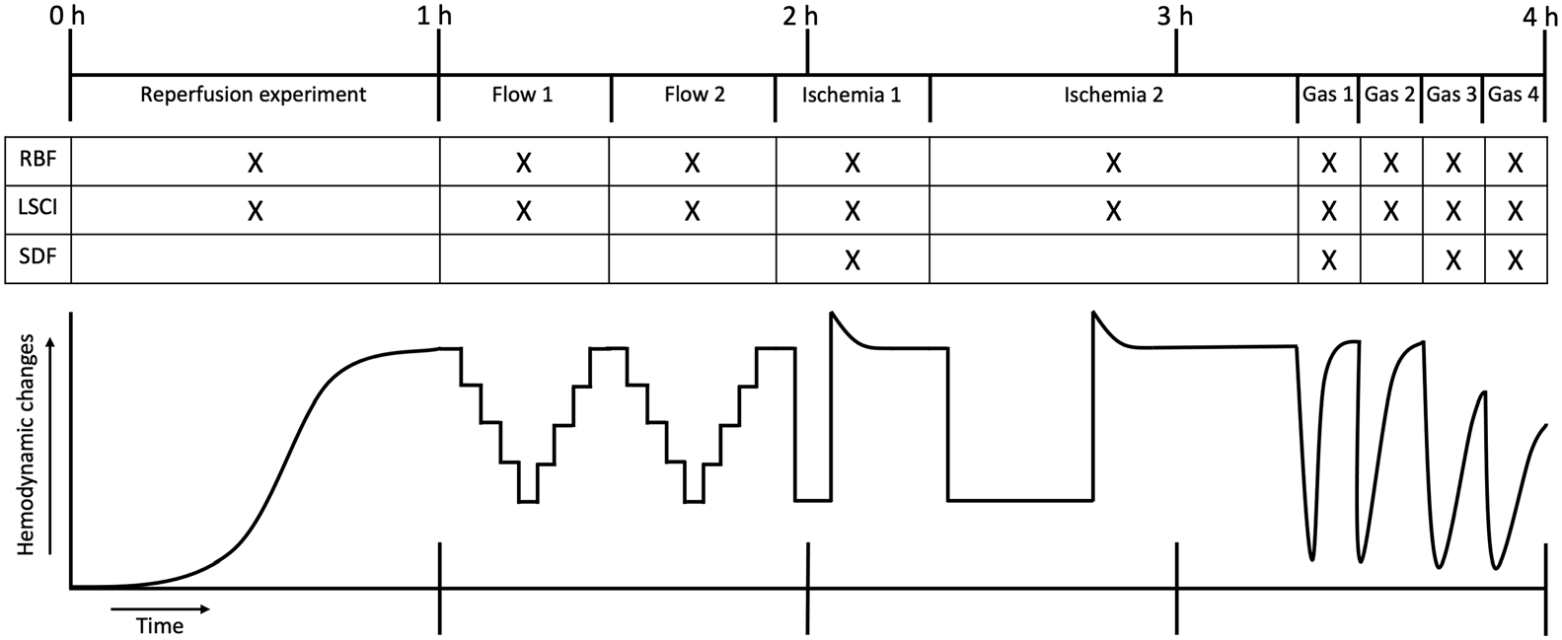
A time-scaled overview of the total experiment. The experiment was 4 h in total and can globally be split up into four different experiments; the reperfusion of the kidney, a stepwise modulation of arterial renal flow, the induction of a local ischemic area, and the gas bubble infusion of $O_{2}$, carbogen (95% $O_{2}$ and 5% ${\mathrm{CO}}_{2}$), room air, and $N_{2}$. The table indicates the techniques that have been used to monitor the hemodynamic changes (RBF, renal blood flow; LSCI, laser speckle contrast imaging; and SDF, sidestream dark-field imaging). The graph represents the hemodynamic changes as measured by the arterial RBF probe [RBF (ml/min)].

We have designed a set of four hemodynamic experiments to study the LSCI as a tool for organ perfusion measurement (Fig. 2). During all experiments, the temperature, pressure, the (arterial) RBF, and renal resistance were measured. Subsequently, we measured the cortical perfusion using LSCI. These experiments were designed to examine the power to differentiate between well and poorly perfused tissues over time (Secs. 2.4.1 to 2.4.3) and to analyze the techniques’ ability to distinguish between local perfusion deficits, i.e., spatial resolution, and the speed at which this is detected, i.e., temporal resolution (Secs. 2.4.3 and 2.4.4). These experiments were repeated on five different kidneys. In a separate extra kidney, we have performed RBF, LSCI, and sidestream dark-field (SDF) imaging simultaneously while performing a repeated local ischemia and three gas bolus injection (Sec. 2.5).

#### 2.4.1 Reperfusion experiment

Validating the relation between the RBF and LSCI values is crucial for clinical use. Apart from the resemblance with the reperfusion during a kidney transplant, this experiment could provide us with insights about the correlation between RBF and LSCI values in low- and high-flow values at the beginning and end of the experiment, respectively. After HMP and a cold flush with 500 ml of 0.9% NaCl, the kidney was installed in the NMP organ chamber, warming up for 60 min with a pressure of 85 mmHg and a sinusoidal flow with a frequency of 60 Hz to mimic a physiological situation. During this hour, the kidney warms-up from 4°C to 37°C.

#### 2.4.2 Flow experiment

Similar to the reperfusion experiment (Sec. 2.4.1), this experiment could give us insight in the correlation between RBF and LSCI values in a step-wise and controlled manner. For this experiment, the flow was switched from sinusoidal perfusion to constant flow to guarantee a stable linear flow without interference of the sinusoidal pattern. The flow was set at a rate of 200  ml/min. The experiment was started at 150  ml/min in case the kidney did not reach the flow of 200  ml/min after the warm-up phase. The flow was decreased by steps of 50  ml/min every 4 min. The flow was subsequently increased with steps of 50  ml/min to the starting level when a flow of 50  ml/min was reached. This experiment was performed twice, consecutively on every kidney.

#### 2.4.3 Local ischemia

We could evaluate the ability to distinguish between well and non-perfused tissues within the FOV by inducing a local ischemic area. The speed at which the large local perfusion difference can be visualized gives an indication of the added clinical value in case of an unwanted local perfusion deficit during a kidney transplant. A catheter (4F arterial Embolectomy Catheter, Edward Lifescience, Irvine, United States) was inserted in the renal artery before NMP and sutured in the inferior bifurcation of the renal artery. The catheter was inflated inducing local ischemia in one part of the kidney. The ischemia was induced twice. The first time, a short 5-min warm ischemic period was followed by a 10-min recovery time. The second time, a long 15-min warm ischemic period was followed by a 40-min recovery time.

#### 2.4.4 Gas-bubble infusion

Although local ischemia (Sec. 2.4.3) causes a large perfusion deficit, this experiment tests the ability to distinguish between these areas of local reperfusion and to observe these in time. The infusion of an arterial gas bolus induces a short complete ischemia that is followed by small areas of local reperfusion with eventually complete reperfusion of the kidney. The gas forms an embolism in the blood vessel. These emboli prevent blood from passing through and thereby hamper the perfusion. When the gas dissolves in the perfusion medium, the embolism disappears and perfusion reappears. The speed at which this occurs depends on the injected gas and its relative solubility (i.e., $O_{2}$ and ${\mathrm{CO}}_{2}$, dissolve faster than $N_{2}$). As the last experiment, 4 ml of the respective gasses were injected into the arterial line. First with $O_{2}$, subsequently followed every 10 min by carbogen (95% $O_{2}$ and 5% ${\mathrm{CO}}_{2}$), room-air, and $N_{2}$.

### 2.5 Sidestream Dark-Field Imaging Experiments

SDF imaging can track the movement of individual RBCs enabling quantitative blood flow measurements and the detection of subtle microvascular changes.^17^^–^^19^ SDF imaging is a contact method with a relatively small FOV of $∼1\;{\mathrm{mm}}^{2}$. The device has a total magnification of 750×. The penetration depth is around 750  μm. The small FOV in combination with a shallow penetration depth and the fact that it is a contact method makes SDF imaging less than ideal for the visualization or the RCM. The green light emitted by the system scatters through the tissue and is absorbed by the hemoglobin in the RBCs resulting in dark RBCs in contrast to background tissue. Using SDF imaging as a quantitative measure of blood flow allows us to compare the RBF and LSCI with SDF. However, it must be noted that RBF measures both cortical and medullar blood flow, whereas LSCI and SDF measure only the RCM with the difference of being full field (LSCI) versus a small FOV (SDF). The SDF microscope (MicroScan^®^ Video Microscope System, MicroScan BV, Amsterdam, The Netherlands) was held in place using a tripod connected to a laboratory table to minimize movement artifacts. The tripod has X- and Y-axis precision adjustment screws to place the microscope perpendicular to the renal cortex without inducing pressure artifacts. The tip of the SDF microscope was covered with a plastic cap. Images were recorded at 10  frames/s with a 720×576  pixel resolution. The video signal was digitalized using an S-VHS to USB frame grabber and stored on a computer for further offline processing. The SDF microscope uses pulsating green light-emitting diodes that are placed around the charge-coupled device at the tip of the microscope. The data were analyzed using custom software (Matlab, Mathworks, Natick, Massachusetts) that calculated the mean pixel intensity (MPI) within the whole frame by taking the average pixel intensity. The MPI is a measure of the number of RBCs. As the number of RBCs within the frame increase, the images darken hence the MPI is a relative measure for the number of RBCs (Video 3). The region of interest for the laser speckle perfusion units (LSPU) was placed 1 cm away from the SDF camera.

The renal capsule had to be locally removed at the tip of the SDF microscope to be able to image the RCM during this part of the experiments. SDF imaging, RBF measurements, and LSCI were performed simultaneously in only one kidney due to the complex nature of imaging the RCM using SDF imaging. Five consecutive repetitions of the short local ischemia experiments were executed followed three gas injections with oxygen, one with room air, and one with nitrogen, respectively.

### 2.6 Data Analysis

Data are presented as mean±SD, unless stated otherwise. The correlations between LSCI in LSPU (A.U.) and RBF (ml/min), and SDF in MPI (A.U.) were calculated using a coefficient of determination, $R^{2}$. Applicable parametrical paired tests were used. A p-value of <0.05 was considered statistically significant. The experiments described in Sec. 2.4 were repeated five times to rule out unique events.

## 3 Results

The average weight of the six slaughterhouse kidneys was 338.1±24.0  g.

### 3.1 Reperfusion Experiment

**Fig. 3:**
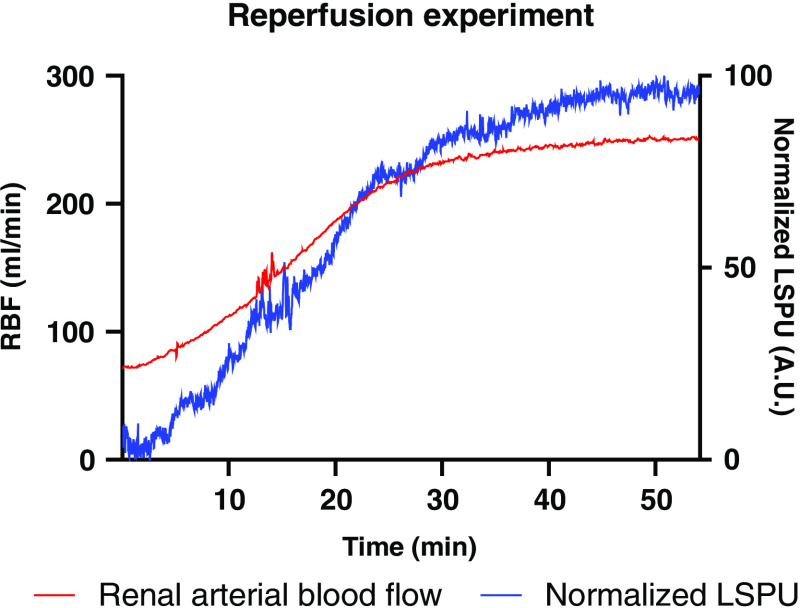
Graph of average laser speckle units perfusion (LSPU) (A.U.) and average renal arterial blood flow (RBF) (ml/min) of five kidneys during the first hour of NMP after the HMP is captured in the reperfusion experiment. The kidney slowly warms-up from 4°C to 37°C with a resulting increase RBF.

The reperfusion experiment was performed five times in five kidneys. During the hour, all kidneys warmed-up to 37°C, which led to an increase in RCM (Fig. 3.). This experiment showed resemblance with the reperfusion during a kidney transplantation. The correlation between the normalized LSPU (A.U.) and the RBF (ml/min) was $R^{2}=0.94\pm 0.02$ (p<0.0001). The good correlation might be explained by the tendency of a kidney to allocate blood to the cortex first, which is the flow that we measure using LSCI.

### 3.2 Flow Experiment

**Fig. 4:**
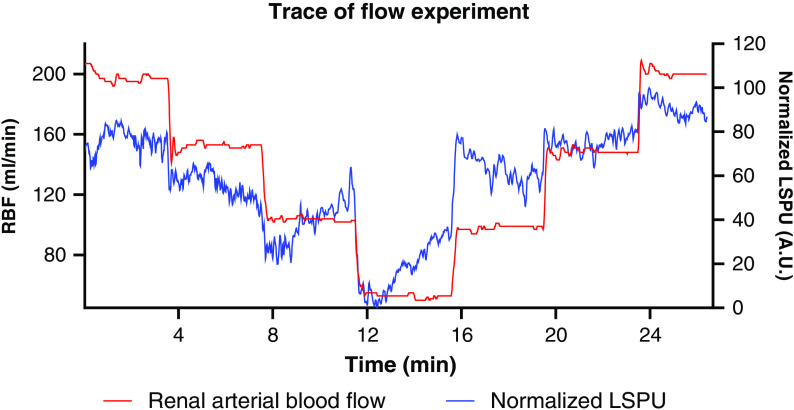
A typical flow experiment trace with normalized LSPU in A.U. and RBF in ml/min. The flow was decreased by steps of 50  ml/min every 4 min and subsequently increased with steps of 50  ml/min to the starting level when a flow of 50  ml/min was reached.

The flow experiment was performed 10 times in five kidneys. The $R^{2}$ of the normalized LSPU (A.U.) and RBF (ml/min) was 0.59±0.31 (p>0.05). A change in total RBF was not followed by a similar change in the cortex (i.e., RCM) resulting in a moderate correlation. As seen in Fig. 4, there seems to be a hemodynamic response, redirecting flow to the cortex. There was an apparent hemodynamic response when the RBF was decreased in a low flow state (∼100  ml/min). When the RBF was increased, a classical short-term reperfusion overshoot was observed.

### 3.3 Local Ischemia

**Fig. 5:**
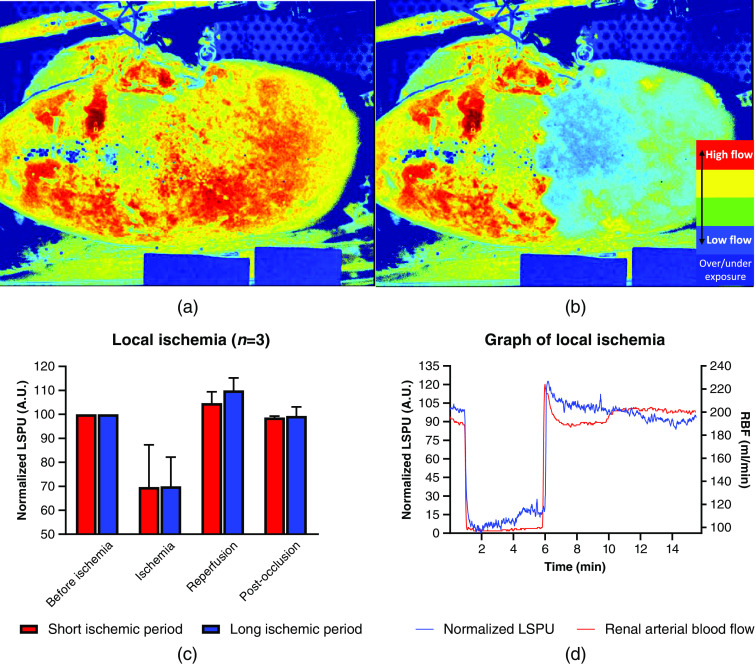
The inflation of a balloon catheter in an arterial bifurcation induces a local ischemic area. The local ischemia was induced consecutively for a short (5 min) and long (15 min) period in each kidney. (a) Pseudo-color image of the RCM before induction of local ischemia made using LSCI. (b) Pseudo-color images of the RCM while local ischemia is induced made using LSCI. (c) Relative drop of the RCM measured in LSPU in A.U. for the short and long ischemic period. The results are normalized on the average of the baseline period. (d) A typical local ischemia trace measured by RBF (ml/min) and the RCM in normalized LSPU (A.U.) where the LSPU is normalized on the min/max of the whole local ischemia experiment (Video 1, MP4, 8.2 MB [URL: https://doi.org/10.1117/1.JBO.26.5.056004.1]).

The local ischemia experiment was performed on five kidneys. The experiment failed for two kidneys; one due to malfunctioning of the balloon catheter and the other one due to the appearance of the ischemic area on the posterior side of the kidney. Typical images are shown in Figs. 5(a) and 5(b). The data are depicted in bar graphs in Fig 5(c) and a typical trace of an ischemic region of interest is displayed in Fig. 5(d). The results of the short (5 min) and long (15 min) ischemic period are found in Table 1. The short and long ischemic period did not show any significant differences. The baseline is the average of the time period before the induction of the local ischemia. The ischemia is the average of the ischemic period. The reperfusion is the maximum value directly after the release of the local ischemia and the postocclusion is the average of the time after the reperfusion. The LSPU values are normalized compared to the baseline.

**Table 1:** Relative change in LSPU normalized compared to the baseline for the ischemia, reperfusion, and post-occlusion period for both the short and long ischemic period, respectively. The $R^{2}$ value is calculated between the RBF in ml/min and the LSPU in A.U. for the short and long ischemic period.

	Short ischemic period	Long ischemic period
Baseline	100±0%	100±0%
Ischemia	70±14%	70±10%
Reperfusion	105±4%	110±4%
Post-occlusion	99±0%	100±3%
$R^{2}$ of LSPU (A.U.) and RBF (ml/min)	0.90±0.03 (p<0.0001)	0.81±0.08 (p<0.0001)

### 3.4 Gas-Bubble Infusion

**Fig. 6:**
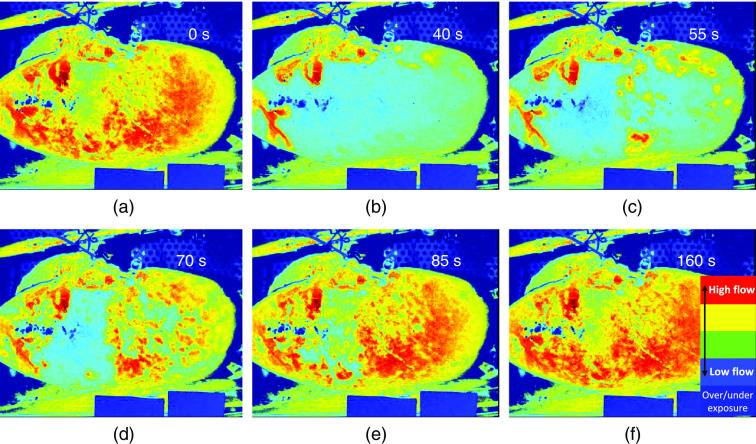
Pseudo-color images of the RCM during a gas-bubble (oxygen) injection made using LSCI. The injected oxygen forms a gas embolism in the blood vessels. These emboli prevent blood from passing through and thereby hamper the perfusion. When the gas dissolves in the perfusion medium, the embolism disappears and perfusion reappears. (a) Before oxygen injection, (b) directly after oxygen injection, (c)–(f) slow, local return of the RCM (Video 2, MP4, 9.2 MB [URL: https://doi.org/10.1117/1.JBO.26.5.056004.2]).

**Fig. 7:**
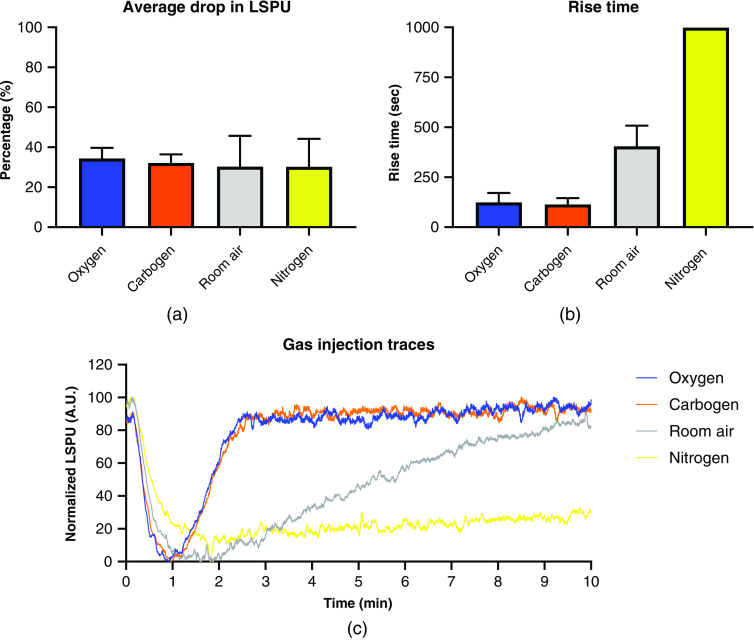
The infusion of a gas bolus induces a short complete ischemia that is followed by growing areas of local reperfusion. (a) The average percentage drop in LSPU in A.U. after injection of the respective gas. (b) The recovery time of the LSPU to pre-injection perfusion level in seconds (c) Typical LSPU traces of the respective gasses of one kidney.

The gas-bubble infusion was performed once on five kidneys and characterized by a slow, local return of RCM, as depicted in Fig. 6. The data can be found in Table 2 where the relative drop in LSPU is calculated in relation to baseline level. The rise time is defined as the time it takes for the LSPU to return to the baseline level. This was longer than 600 s for nitrogen and thus the exact rise time could not be measured. The $R^{2}$ was calculated with the LSPU (A.U.) and the RBF (ml/min). The data are visualized in bar graphs in Figs. 7(a) and 7(b) for the average drop (%) and rise time (s), respectively. Figure 7(c) displays typical LSCI traces for this experiment in one kidney.

**Table 2:** The relative drop in LSPU in A.U. compared to baseline after the injection of oxygen, carbogen, room air, and nitrogen. The time to baseline is the total time required for the LSPU to reach baseline. This was longer than 600 s for nitrogen and thus was not measured. The $R^{2}$ value is calculated between the RBF in ml/min and the LSPU in A.U.

	Oxygen	Carbogen	Room air	Nitrogen
Relative drop in LSPU	34±5.0%	32±4.0%	30±4.0%	30±12%
Time to baseline (s)	124.1±42.1	113.9±28.1	404.5±92.5	>600
$R^{2}$ of LSPU (A.U.) and RBF (ml/min)	0.81±0.06 (p<0.0001)	0.74±0.11 (p<0.0001)	0.59±0.18 (p<0.0001)	0.36±0.23 (p<0.0001)

### 3.5 Sidestream Dark-Field Imaging Comparison

**Fig. 8:**
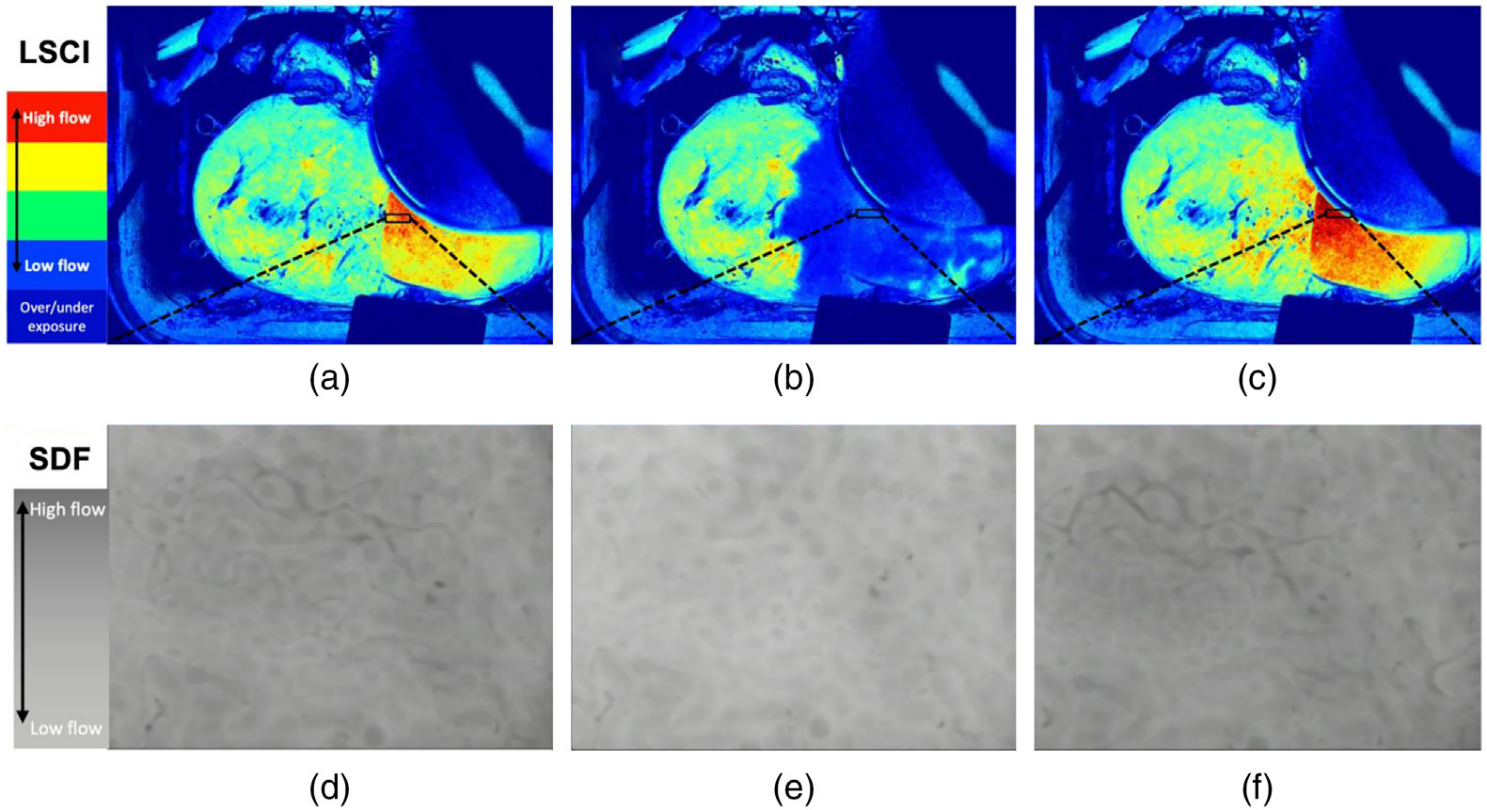
Pseudo-color LSCI images and SDF images of a local ischemia induced by inflation of an arterial balloon catheter. The LSCI and SDF images were recorded simultaneously during the experiment.– Pseudo-color LSCI images of (a) pre-local ischemia, (b) during local ischemia, and (c) reperfusion after local ischemia. (d)–(f) SDF images of the corresponding LSCI images, darker SFD images indicate more RBCs present in the FOV, i.e., better perfusion (Video 3, MP4, 11.6 MB [URL: https://doi.org/10.1117/1.JBO.26.5.056004.3]).

The results of the local ischemia, oxygen, room air, and nitrogen injection are shown in Table 3. All experiments were performed on the same kidney. A representative example of the LSCI pseudo-color images and the corresponding SDF images are shown in Fig. 8 and Video 3. SDF-LSPU showed the best overall correlation compared to the LSPU-RBF and SDF-RBF $R^{2}$-values.

**Table 3:** The $R^{2}$ values of the SDF, the LSPU, and the total RBF during the SDF imaging experiment. The local ischemia, oxygen, room air, and nitrogen injection were performed n times on the same kidney. The experiment consisted of five short consecutive local ischemic periods followed by three oxygen, one room air, and one nitrogen injection.

	n	$R^{2}$ of SDF-LSPU	$R^{2}$ of LSPU-RBF	$R^{2}$ of SDF-RBF
Local Ischemia	5	0.85*	0.87*	0.81*
Oxygen	3	0.86*	0.41*	0.38*
Room air	1	0.83*	0.66*	0.36*
Nitrogen	1	0.60*	0.22*	0.03*

Note: LSPU, laser speckle perfusion units; RBF, renal blood flow.

* p<0.0001.

## 4 Discussion

We report on the visualization of RCM using *ex vivo* perfused human-sized porcine slaughterhouse kidneys in various models of ischemia/reperfusion. The local reperfusion experiments demonstrated a high correlation between the LSCI and SDF, superior to LSCI correlations with RBF. The good correlation between LSCI and SDF emphasizes the high temporal and spatial resolution of LSCI in its ability to visualize RCM. LSCI not only display a clear distinction between perfused and non-perfused tissues, it also tracks the transient ischemia, induced by injection of gasses with different absorption characteristics, in a real-time manner. Yet, the effect of the monitoring of near-surface cortical microperfusion on clinical decision making should be further investigated in a clinical trial.

The reperfusion experiment displayed a high correlation between the RBF and LSCI indicating that the reperfusion after ischemia during renal transplantation could be monitored using LSCI. This has the advantage over conventional intrarenal probes that it can detect local perfusion deficits in an early stage. The slight difference between RBF and LSCI could potentially be explained by the kidney’s redistribution mechanism^20^ since the perfusion of the medulla and cortex are a dynamic process and are influenced by hemodynamic factors. Our data have indicated that a stable RBF does equate a stable cortical perfusion, as the perfusion of the cortex and the medulla can change over time and independent of each other (e.g., when the RBF does not change, LSCI can still detect the local perfusion deficits).

During the flow experiment with a stepwise change in total RBF, LSCI displayed only a moderate correlation with total RBF. We hypothesize that the functional tissue in the cortex is preserved at the expense of a reduction in flow to the medulla in a response to a decrease in RBF. This would result in a redirection of flow to the cortex (i.e., RCM).^20^ In our data, when the RBF was decreased, RCM gradually increased after each drop in total RBF as a result of autoregulation in favor of RCM. However, when the RBF was increased in low-flow situations, the contrary was witnessed.

The local ischemia was immediately visible on the real-time live feed. The experiment has a good correlation for both the short and long ischemic period, which is comparable to the correlation found during the LSCI-SDF comparison. This can be explained by the fact that there is no blood to redistribute, thus the decrease in flow is proportional to the RBF. It instantly displays a distinction between well- and non-perfused tissues, whereas visible tissue discoloration takes a prolonged amount of time. This fast and accurate assessment of the RCM by LSCI has potential clinical impact. For example, Hoffman et al.^9^ reported on a perfusion deficit imperceptible to the human eye that could be restored by repositioning the organ in the iliac fossa. LSCI’s ability to transiently track the infusion of different gasses with different absorption characteristics demonstrates the high spatial and temporal resolution. This is demonstrated by the good correlation between LSCI-SDF and a poor correlation between LSCI-RBF. During the relatively longer period before nitrogen is dissolved in the blood, RBF slowly restores while the cortex takes more time to restore to the full blood flow. This results in a relatively poor correlation, stressing the importance of the use of LSCI.

As mentioned before, SDF imaging directly visualizes individual RBCs. Comparing LSCI to both SDF and RBF gives us valuable information about the perfusion measured using LSCI. However, SDF imaging of a kidney is tedious and required removal of the renal capsule making it unsuitable for clinical practice. The good correlations between LSCI and SDF thus indicate that LSCI can give valuable information with the advantage of being a non-contact and full-field imaging method.

An important clinical need in which LSCI can easily be implemented is organ transplantation. Since prolonged anastomosis time is detrimental for organ quality,^21^ fast and easy visualization of the near-surface RCM could help improve transplantation outcome, especially since there is a relation between the early intraoperative state of microperfusion and postoperative outcomes.^1^^–^^4^ We hypothesize that the visualization of ischemic areas and vascular obstructions without delay directly after reperfusion of the organ could assist the surgeon in clinical decision making. Yet, this has to be further explored in clinical trials. This intraoperative imaging has the potential to reduce reoperation rates compared to the current clinical standard with postoperative imaging such as duplex sonography. By directly showing the surgeon if and where there is a perfusion deficit, counteractive measures can be taken. This does not only account for transplant surgery but for any kind of surgery where whole organ perfusion is of interest.

The use of LSCI has already been described in rat kidneys,^22^^–^^28^ but literature on its use on human-sized kidneys is still lacking. Clinical application would be feasible since LSCI has already been used in a clinical setting.^15^

One of the main challenges that has to be overcome before LSCI can be implemented into clinical practice is movement artifacts.^29^ For these experiments, we have fixated the kidney using pipette tips to eliminate the possible effect of movement. However, *in vivo*, the kidney will be subject to movement as a result of respiratory movement and pulsations of the heart during transplantation. Others have tried to overcome this using fiducial markers.^30^^,^^31^ This solution is not desirable for kidney transplantations due to the invasive aspect of attaching the fiducial marker. Another potential limitation is the shallow penetration depth for LSCI of roughly 0.4 to 1 mm depending on the wavelength.^32^^,^^33^ Nevertheless, this does not limit the use of LSCI, since our data show that ischemia is directly detectable in the RCM.

## 5 Conclusion

In the setting with *ex vivo* machine perfused human-sized porcine kidneys, LSCI was capable of detecting local changes in RCM with a high spatial and temporal resolution. In various settings of local ischemia, LSCI correlated well with SDF imaging. However, LSCI does not always fully correlate with the total RBF due heterogeneity of blood flow between the medullar and cortical microcirculation underlining the added value over conventional arterial flow sensors. The implementation of LSCI during transplant surgery could help with the early establishment of an appropriate treatment plan directly after reperfusion of the organ.
